# Regional analysis of myelin basic protein across postnatal brain development of C57BL/6J mice

**DOI:** 10.3389/fnana.2025.1535745

**Published:** 2025-03-06

**Authors:** Siddhi S. Ozarkar, Ridthi K. R. Patel, Tasmai Vulli, Carlee A. Friar, Alain C. Burette, Benjamin D. Philpot

**Affiliations:** ^1^Neuroscience Center, University of North Carolina at Chapel Hill, Chapel Hill, NC, United States; ^2^Department of Cell Biology and Physiology, University of North Carolina at Chapel Hill, Chapel Hill, NC, United States; ^3^Carolina Institute for Developmental Disabilities, University of North Carolina at Chapel Hill, Chapel Hill, NC, United States

**Keywords:** myelin, myelin basic protein, postnatal development, white matter, myelin-associated glycoprotein

## Abstract

Healthy brain development hinges on proper myelination, with disruption contributing to a wide array of neurological disorders. Immunohistochemical analysis of myelin basic protein (MBP) is a fundamental technique for investigating myelination and related disorders. However, despite decades of MBP research, detailed accounts of normal MBP progression in the developing mouse brain have been lacking. This study aims to address this gap by providing a detailed spatiotemporal account of MBP distribution across 13 developmental ages from postnatal day 2 to 60. We used an optimized immunohistochemistry protocol to overcome the challenges of myelin’s unique lipid-rich composition, enabling more consistent staining across diverse brain structures and developmental stages, offering a robust baseline for typical myelination patterns, and enabling comparisons with pathological models. To support and potentially accelerate research into myelination disorders, we have made >1,400 high-resolution micrographs accessible online under the Creative Commons license.

## 1 Introduction

Myelination, the process by which oligodendrocytes wrap axons in a multilayered, lipid-rich membrane, is fundamental to brain development and function (Flechsig, 1901; Kinney et al., 1988; Nave and Werner, 2014; Monje, 2018; Stadelmann et al., 2019). The myelin sheath serves three critical functions: it enhances signal transmission through saltatory conduction (Hartline and Colman, 2007), provides physical and chemical protection for axons (Nave, 2010a,b), and delivers essential metabolic support for long-term neuronal health (Funfschilling et al., 2012; Lee et al., 2012). Unsurprisingly, proper myelination is essential for many brain functions, from basic motor control and coordination to complex cognitive processes (Kato et al., 2020; Khelfaoui et al., 2024). Consequently, disruptions in myelination are associated with numerous developmental and cognitive disorders (Fields, 2008; Khelfaoui et al., 2024).

The study of myelination spans over a century, beginning with Paul Flechsig's pioneering work mapping myelin development in the human brain using hematoxylin/eosin staining (Flechsig, 1901). This was soon followed by Vogt and Vogt, who established myeloarchitectonic research with the refinement of specific myelin staining techniques (Vogt and Vogt, 1919). The field advanced significantly in the 1980s with the introduction of immunohistochemical methods targeting key myelin components, particularly through the work of Sternberger et al. (1979), Itoyama et al. (1980), and Bjelke and Seiger (1989), among others (Sternberger et al., 1979; Itoyama et al., 1980; Bjelke and Seiger, 1989).

Recent technological advances have introduced new tools for studying myelination. Magnetic resonance microscopy and magnetic resonance histology enable non-invasive visualization of white matter across the entire mouse brain (Rahman et al., 2023). However, their spatial resolution remains limited by the size disparity between axons (~1 μm diameter) and imaging voxels (tens of μm) (Petiet et al., 2008; Wang et al., 2019). While emerging techniques like synchrotron x-ray microcomputed tomography and light sheet fluorescence microscopy show promise for high-resolution imaging, immunohistochemistry for myelin basic protein (MBP) remains the gold standard for detailed myelination studies in mice.

MBP, comprising 30% of total CNS myelin protein, is crucial for both myelin formation and maintenance (Readhead et al., 1990; Shine et al., 1992; Bakhti et al., 2014). Its importance is demonstrated by shiverer mice, which lack MBP and fail to form compact myelin sheaths (Inoue et al., 1981; Roach et al., 1985). MBP’s continued incorporation is crucial for maintaining myelin integrity in the mature brain (Meschkat et al., 2022). Given its crucial role and prevalence, MBP is a standard marker for visualizing the myelin sheath.

Although MBP expression in the rodent brain has been extensively studied (Jacque et al., 1985; Rozeik and Von Keyserlingk, 1987; Schwab and Schnell, 1989; Hamano et al., 1996; Collins et al., 2018), comprehensive analysis of its spatial and temporal distribution across the developing rodent central nervous system is still scarce. Existing studies often focus on specific brain areas and developmental ages, limiting cross-regional and -temporal comparisons. A notable exception is the work by Bjelke and Seiger (1989), which carefully describes MBP immunostaining in the developing rat brain. However, the study is limited to four developmental ages, and due to limitations imposed by traditional publishing formats at the time, their report was confined to seven figures with microphotography and drawings, severely constraining the details they could share. Like many early investigations, this study also focused on the rat brain. Although myelination in rats and mice follows a similar trajectory, subtle yet significant differences exist in their developmental timelines (Clancy et al., 2001). These timing variations are crucial because myelination can progress rapidly within specific brain regions. As a result, even minor interspecies discrepancies can lead to substantial differences in the onset and progression of myelination. This can complicate the direct application of findings from rat models to understanding myelination patterns in mice, highlighting the need for detailed studies specifically in mice.

The technical limitations that constrained earlier studies have been largely overcome. Advances in imaging technology and data sharing now enable the acquisition and dissemination of more comprehensive datasets with higher spatiotemporal resolution, paving the way for detailed investigations. This study leverages high-throughput microscopy to offer a detailed view of MBP distribution in the developing mouse brain, spanning 13 developmental stages with fiber-level resolution. We make over 1,400 micrographs (0.3 μm/pixel) publicly available through the BioImage Archive (accession S-BIAD1483, DOI: 10.6019/S-BIAD1483). These images can also be viewed and interacted with directly on the Image Data Resource (IDR) website^1^ (Williams et al., 2017) under accession number idr0166.

This dataset can serve as a baseline of normal myelination in the C57BL/6 J mouse brain, a commonly studied mouse strain, allowing comparison with white matter disease models. Additionally, it contributes to the growing pool of large-scale, open-access datasets, supporting the trend toward data reanalysis in neuroscience. By offering this detailed resource, we aim to help research into myelination processes and white matter pathologies, potentially leading to improved understanding and treatment of related disorders.

## 2 Materials and methods

### 2.1 Antibodies

To identify MBP, we used a rat monoclonal antibody (Abcam Cat#ab7349, RRID: AB_305869) raised against the full-length protein corresponding to cow MBP. This antibody binds to a region defined by amino acids 82-87 (DENPVV). The specificity of this antibody was confirmed using oligodendrocytes and tissue from Shiverer mice, which lack MBP expression (Zuchero et al., 2015).

To identify myelin-associated glycoprotein (MAG), we used a rabbit monoclonal antibody (Cell Signaling Technology Cat# 9043, RRID:AB_2665480) raised against a synthetic peptide corresponding to residues surrounding Arg605 of human MAG protein. Western blot analyses confirmed the specificity of the antibody, revealing a distinct band at approximately 100 kDa, consistent with the expected molecular weight of MAG.

To identify phosphorylated neurofilament H, we used a mouse monoclonal antibody (Millipore Cat# NE1022-100UL, RRID:AB_2043448) raised against homogenized hypothalamic tissue from Fischer 344 rats (Xiao and Monteiro, 1994). This antibody strongly recognizes the ~180-200 kDa phosphorylated neurofilament H protein and, to a lesser extent, phosphorylated neurofilament M protein.

### 2.2 Animals

All animal procedures followed institutional and NIH guidelines. We used C57BL/6J mice from postnatal day (P)2 to P60. To control for the potential effects of maternal care and nutrition on development, litter sizes were standardized to 5-7 pups. Table 1 provides detailed information about the number of mice used at each age, their sex distribution, and litter information.

**Table 1:** Summary of the age, sex, and number of mice used in this study.

Age	Sex	Litters
P5	3 M	3
P8	3 F	3
P10	1 M, 2 F	2
P12	2 M, 1 F	3
P16	2 M, 1 F	3
P20	1 M, 2F	3
P23	1 M, 2 F	3
P28	2 M, 1 F	3
P30	1 M, 2 F	3
P45	2 M, 1 F	3
P60	3 F	3

### 2.3 Tissue preparation

Mice were deeply anesthetized using sodium pentobarbital (60 mg/kg, administered intraperitoneally). We then performed intracardiac perfusion with phosphate-buffered saline (PBS, 0.1 M, pH 7.3), followed by 10-minute perfusion with 4% freshly depolymerized paraformaldehyde in phosphate buffer (pH 7.3). After extraction, the brains were post-fixed overnight at 4°C in the same fixative solution. Subsequently, we cryoprotected the tissue in 30% sucrose in PBS. Finally, we used a sliding microtome to sagittal section the brains at 50 μm thickness. Sections were stored in a cryopreservative solution (45% PBS, 30% ethylene glycol, 25% glycerol) at −20°C until further processed. At least one of every four sections was processed for enhanced MBP staining and imaging, with images captured no more than 200 μm apart.

### 2.4 Immunohistochemistry

For standard immunofluorescent staining, free-floating sections were rinsed thoroughly in PBS before a 30-minute room temperature block in PBS with 10% fetal bovine serum and 0.2% Triton-X-100 (FBST). Blocked sections were then incubated overnight at room temperature with primary antibodies (MBP at 1:2,000 and MAG at 1:500) diluted in FBST. Following several rinses in PBST (PBS with 0.2% Triton-X-100), sections were incubated overnight at room temperature with Alexa Fluor-conjugated secondary antibodies and DAPI (700 ng/mL) diluted in FBST.

For enhanced immunofluorescent staining, free-floating sections were initially permeabilized with methanol (2 x 15 min in 50% methanol/PBS), followed by a second permeabilization step for 1 hour at 37°C in a solution of 2.3% glycine, 20% DMSO, and 0.2% Triton X-100 in PBS. Sections were then preincubated in 5% DMSO, 0.1% Triton X-100, 1% BSA in PBS for 30 minutes, followed by overnight incubation at 37°C with primary antibodies (MBP at 1:2,000 and MAG at 1:500 or MBP at 1:2,000 and SMI-31 at 1:1,000 diluted in PBS with 5% DMSO, 0.1% Triton X-100, 1% BSA, 0.2% Tween-20, and 1% heparin). Visualization of the primary antibodies was achieved using Alexa Fluor-conjugated secondary antibodies. DAPI counterstaining was employed to highlight nuclei. To assess staining quality across different protocols, sections were examined using Leica STELLARIS 8 FALCON microscopes.

### 2.5 Imaging and figure production

For imaging, we used an Olympus SLIDEVIEW VS200 slide scanner equipped with a SILA 4-line laser combiner (405 nm, 488 nm, 561 nm, and 638 nm) and scrambler unit (Evident Scientific, Inc. Waltham, MA). All brain sections were imaged using a UPLXAPO 20X objective (NA 0.8) and a Hamamatsu ORCA-Fusion camera (2,304 × 2,304), producing 16-bit grayscale images with approximately 0.3 μm/pixel resolution.

We optimized our imaging parameters, adjusting exposure time and laser intensity to capture the full spectrum of MBP staining intensities within the 16-bit scale. This calibration ensured we could accurately detect staining ranging from the faintest signals (such as weakly positive oligodendrocytes at P2) to the most intense (such as white matter tracts in the P60 brain). We kept consistent imaging settings throughout our study. This method facilitates more straightforward comparisons between various brain regions and time points, and it also opens the possibility for others to conduct quantitative analyses. We employed the QuPath software package (RRID: SCR_018257) (Bankhead et al., 2017) for dataset exploration and organization.

We used CorelDRAW Graphics Suite 2024 (Alludo, Ottawa, ON, Canada, RRID: SCR_014235) for image adjustment and figure creation. The intensity of MBP staining exhibited considerable variation across different brain regions and developmental stages. This resulted in raw images with a high dynamic range, making it difficult to fully capture the staining details in figures. For instance, the significant difference in fluorescent intensity between white matter tracts and adjacent gray matter made it impossible to discern subtle staining details in the gray matter.

To improve staining visibility, we adjusted the gamma curve and inverted the images in the figures. This inversion transformation converts dark values to light and vice versa, displaying the gray fluorescence signal on a white background. Such presentation enhances perceptual contrast for readers adapted to a white page in bright conditions, thereby improving the visibility of faint features. The online dataset provides the original, unadjusted high dynamic range images, allowing for independent analyses.

## 3 Results

### 3.1 Improving MBP immunostaining

**Figure 1:**
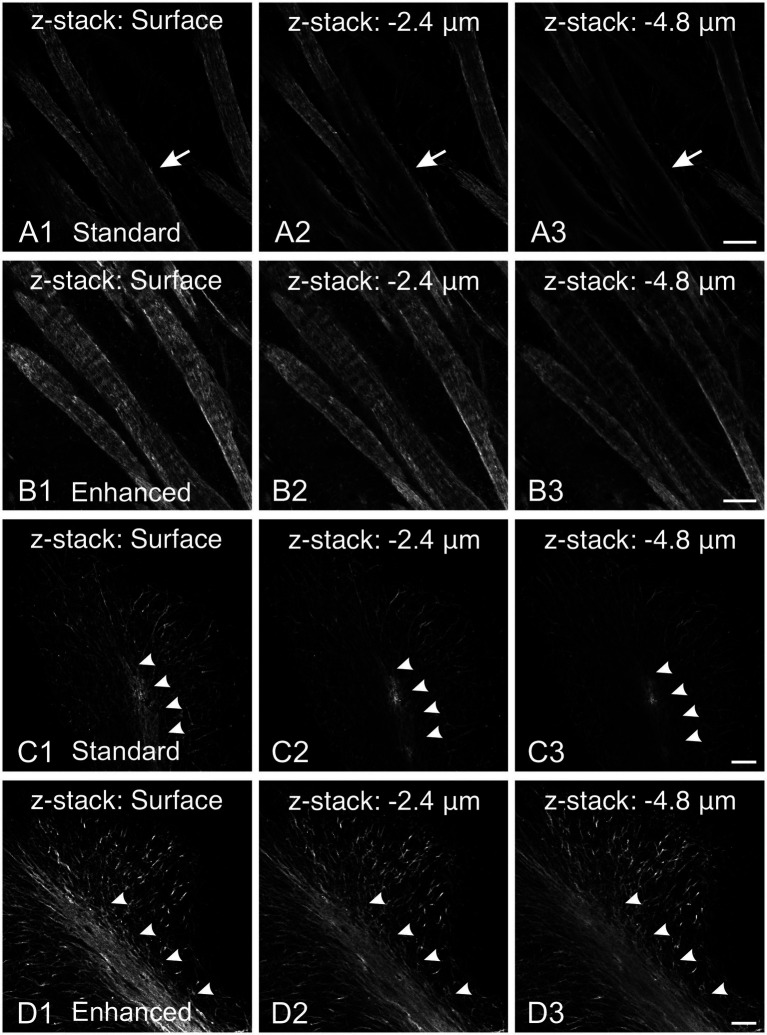
Comparison of MBP staining using standard and enhanced permeabilization protocols. **(A,B)** MBP staining in the striatum using standard Triton X-100 permeabilization protocol **(A)** and enhanced permeabilization protocol **(B)**. With our standard protocol, at the section surface, fascicles show uniform MBP staining from the periphery to their center (**A1**, arrow). However, deeper in the section, MBP staining progressively concentrates at the periphery, leaving most of the axon bundle unstained (**A2,A3**, arrows). In contrast, using the enhanced protocol, MBP staining is more intense and uniform across the section thickness **(B)**. Fiber bundles display more consistent inside-out staining with better-resolved individual fibers. **(C,D)** MBP staining in the cerebellum using standard Triton X-100 permeabilization protocol **(C)** and enhanced permeabilization protocol **(D)**. Using our standard protocol, MBP staining in the arbor vitae (arrowheads) rapidly fades with depth **(C)**. The staining intensity of individual fibers in the cerebellar cortex decreases, but less dramatically. In contrast, using the enhanced permeabilization protocol, MBP staining is more intense and uniform throughout the section thickness **(D)**. Scale bars = 50 μm.

MBP immunostaining is notoriously fickle. Our first MBP staining attempts using a standard Triton X-100 permeabilization protocol yielded inconsistent results across different fiber architectures (Figure 1). MBP labeling was as expected in regions and developmental ages with sparse myelinated fibers, with well-defined labeled fibers extending throughout the section’s thickness. However, MBP staining was impaired in areas with dense fiber bundles and tracts. For example, in the striatum, while individual axons scattered between fascicles stained throughout the section as expected, fascicle staining was compromised (Figure 1A). Surface fascicles (cut during sectioning) showed uniform MBP staining from periphery to center. However, deeper in the section, MBP staining progressively concentrated at the periphery, ultimately leaving most of the axon bundle unstained. This phenomenon was seen in all brain regions with thick axon bundles, including the cerebellum (Figure 1C). These inconsistencies in staining intensity would lead to misinterpretation, especially when comparing myelination patterns across various ages and brain regions. To address this issue, we developed a protocol incorporating a methanol permeabilization step and a combination of permeabilizing agents and solvent (Triton X-100, Tween 20, and dimethyl sulfoxide) in both the permeabilization and antibody solutions. This protocol yielded significant improvements: staining was more sensitive and uniform across section thickness and various structures. Fiber bundles displayed more consistent inside-out staining with better-resolved individual fibers (Figure 1B). Major white matter tracts, such as the cerebellar arbor vitae, also showed more uniform staining (Figure 1D). Due to its increased sensitivity and reduced variability across tissue depths and structures, this enhanced protocol was adopted for all subsequent experiments.

### 3.2 Overall MBP developmental progression

**Figure 2:**
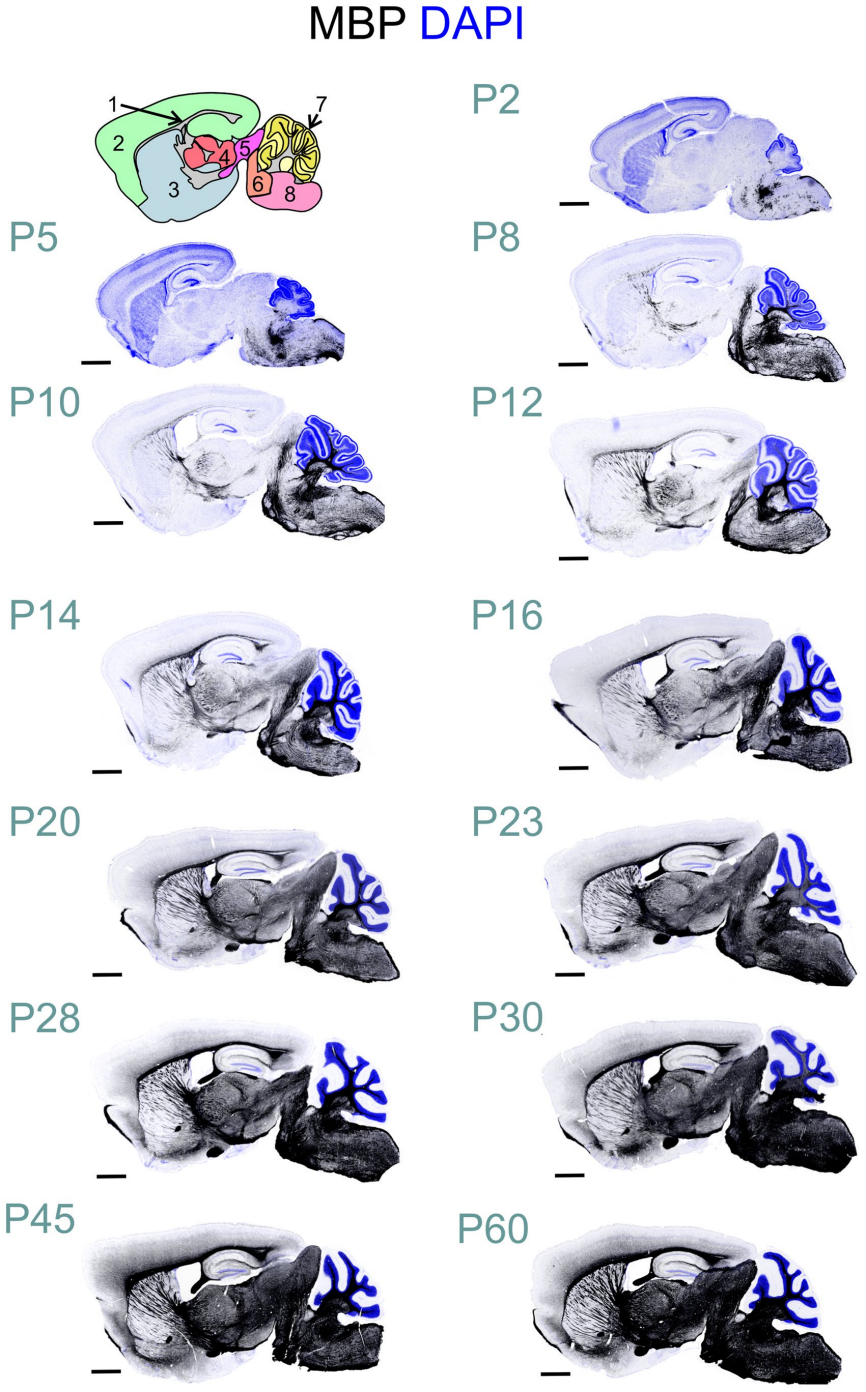
Developmental progression of MBP staining in the mouse brain. Immunofluorescence staining for myelin (MBP; black) and cell nuclei (DAPI, blue) in the mouse brain sections from postnatal day (P)2 to P60 shows a posterior-to-anterior and deep-to-superficial myelination pattern. The hindbrain and cerebellum are the first regions to show MBP positivity, followed by the midbrain, thalamus, basal ganglia, and cerebral cortex. The most rapid MBP staining intensity and distribution increase occurs between P10 and P23. Subsequently, MBP staining continues to intensify and spread throughout the brain, but at a slower pace. Brain regions in cartoon in upper left: 1: Fiber tracts; 2: Cerebral cortex; 3: Cerebral nuclei; 4: Interbrain; 5: Midbrain; 6: Pons; 7: Cerebellar cortex; 8: Medulla. Scale bars = 1 mm.

MBP immunostaining followed the established myelination progression pattern observed in rodents and humans: caudocranial, posterior to anterior, and deep to superficial (Kinney et al., 1988; Deoni et al., 2012). Therefore, the brainstem and cerebellum showed MBP staining before the cerebrum, and within the cerebrum, the basal ganglia and thalami stained before the cerebral cortex (Figure 2). From a whole-brain perspective, we saw the most striking increase in both intensity and distribution of MBP staining between postnatal P10 and P23. However, individual brain regions showed significant variations in their myelination onset and progression rates. After P23, MBP staining continued to increase in intensity and distribution throughout the brain, but at a slower rate.

**Figure 3:**
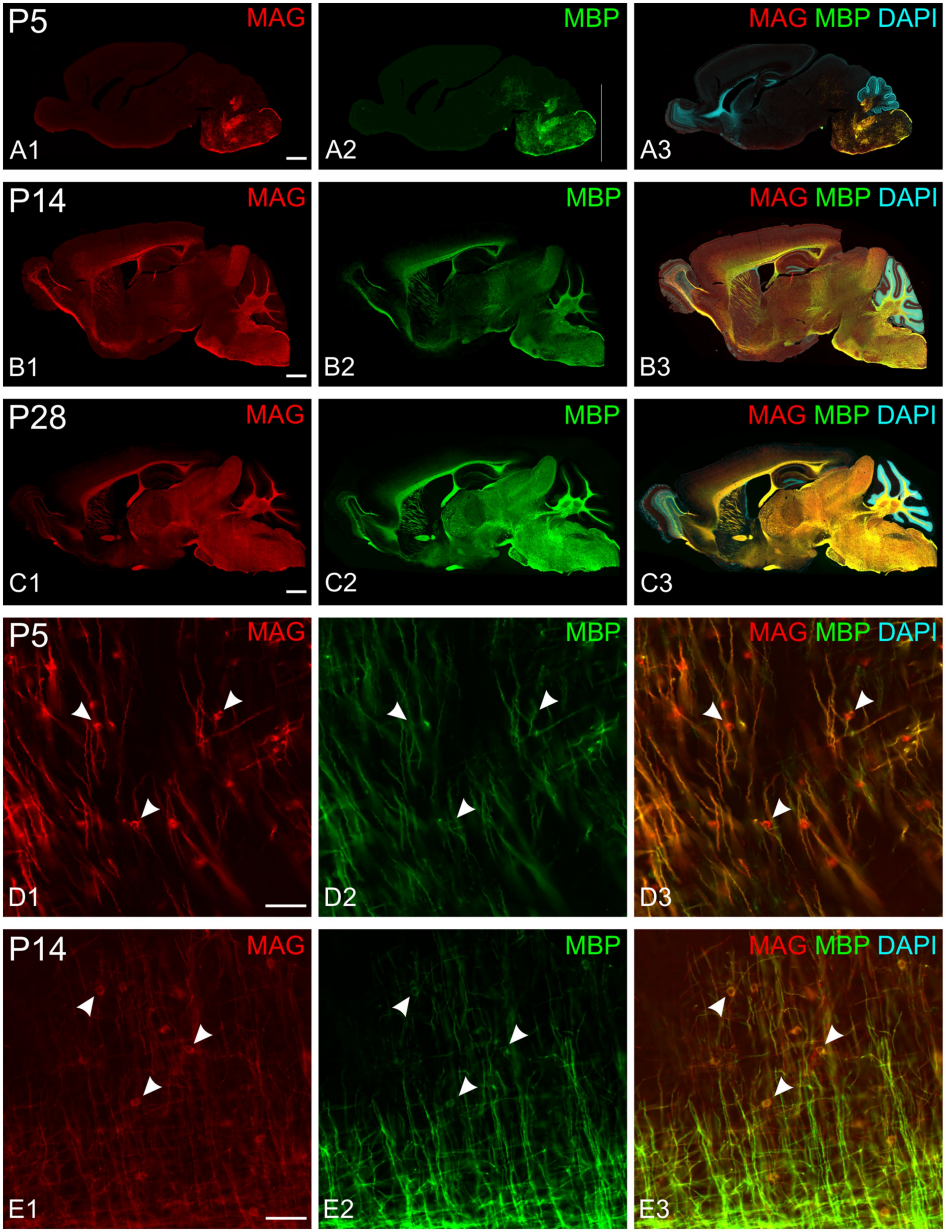
MAG and MBP colocalization in the developing mouse brain. Immunofluorescence staining of MAG (red), MBP (green), and cell nuclei (DAPI, blue) in mouse brain sections at postnatal day 5 (P5; **A,D**), P14 **(B,E)**, and P28 **(C)**. MAG and MBP exhibit similar regional expression patterns across all ages examined. Higher magnification images **(D,E)** show colocalization of MAG and MBP in myelinating fibers. Oligodendrocyte somata are characterized by strong MAG staining but low MBP staining (arrowheads). Scale bars: **(A–C)** = 800 μm; **(D,E)** = 50 μm.

**Figure 4:**
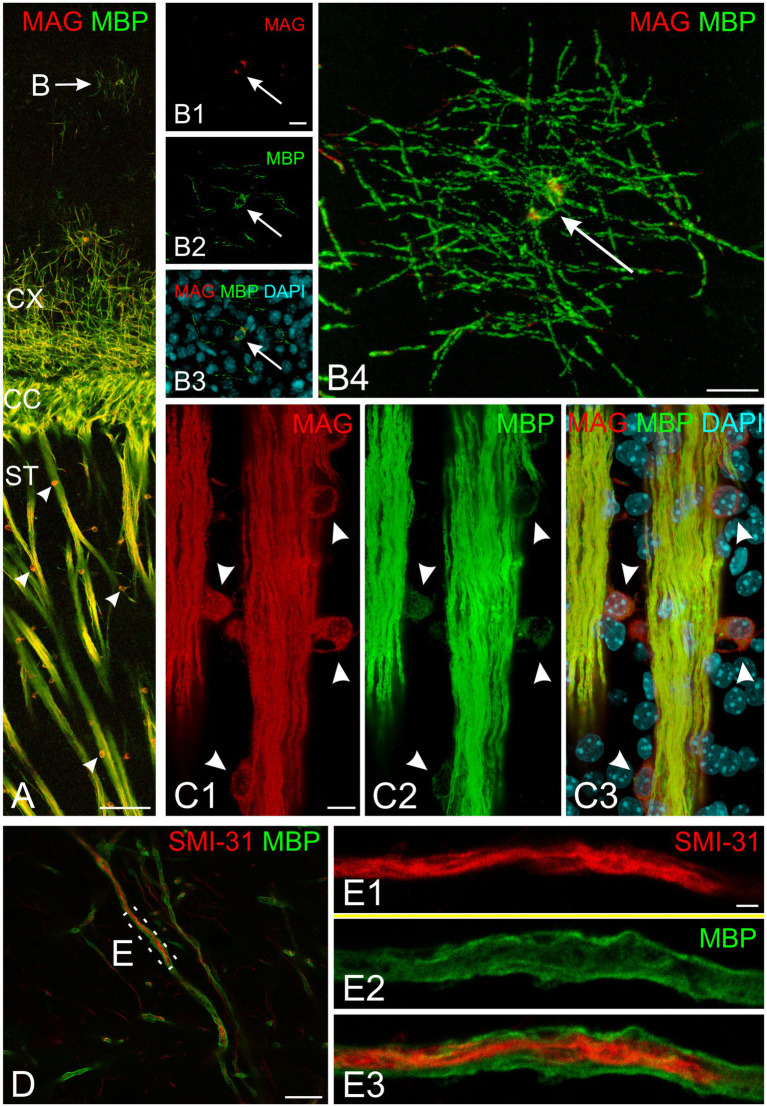
Subcellular organization of myelin proteins in developing mouse brain at P10. **(A)** Overview of MAG (red) and MBP (green) immunofluorescence in the cerebral cortex and striatum. The arrow indicates a cortical oligodendrocyte with MBP-positive radial processes and soma. Arrowheads mark striatum oligodendrocytes showing strong somatic MAG labeling. **(B)** High-magnification confocal images of the cortical oligodendrocyte marked in **(A)** (arrow indicates nucleus). **(B1–B3)** Show a single confocal plane in separate channels and merge; **(B4)** presents the maximum intensity projection of the complete z-stack. Note strong MBP signal in both soma and processes, while MAG localizes predominantly to the soma. **(C)** Detailed view of striatal fiber bundle. Arrowheads indicate oligodendrocyte somata adjacent to fiber bundles. Fiber bundles show strong co-labeling for both MAG and MBP, while oligodendrocyte somata display strong MAG but weak MBP labeling. **(D,E)** STED microscopy of SMI-31 (red) and MBP (green) double labeling. **(D)** Shows myelinated axons in cortical layer 6. **(E)** Presents super-resolution imaging of the boxed region in **(D)**, revealing central SMI-31-positive axon surrounded by MBP-positive myelin sheath. Scale bars, **(A)** = 100 μm, **(B)** = 15 μm, **(C,D)** 10 μm, **(E)** = 2 μm. CC, corpus callosum; CX, cerebral cortex; ST, striatum.

All experiments included labeling for both MAG and MBP. MAG, an abundant myelin protein, plays a crucial role in forming and maintaining myelin sheaths (Quarles, 2007; Pronker et al., 2016). Located on the inner membrane of the myelin sheath, MAG interacts with axonal membrane proteins, effectively anchoring the myelin sheath to the axon. At low magnification, MAG and MBP showed overlapping distribution patterns across the developing brain at all examined ages (Figures 3A–C). During early myelination, at the advancing front of myelination, MBP was present in both oligodendrocyte somata and processes (Figures 3D,E). At this stage, oligodendrocyte processes extended radially from their cell bodies, with their distribution pattern varying by brain region. In the developing cerebral cortex, processes from different oligodendrocytes rarely overlapped, with each cell covering a distinct neuropil domain. In contrast, in the developing corpus callosum, multiple oligodendrocytes covered the same domain. MAG expression, however, was initially restricted primarily to the somata (Figures 3D,E, 4B). As myelination progressed, MBP became increasingly concentrated in processes while decreasing in somata, whereas MAG expression intensified in both processes and somata (Figures 4A–C). This double labeling proved particularly valuable during early myelination stages, helping distinguish between MBP in myelin sheaths versus oligodendrocyte cell bodies and processes. Additionally, we employed STED microscopy using double labeling for MBP and the axonal marker SMI31 (phosphorylated neurofilaments) to confirm the spatial relationship between myelin labeling and axons (Figure 4E).

**Figure 5:**
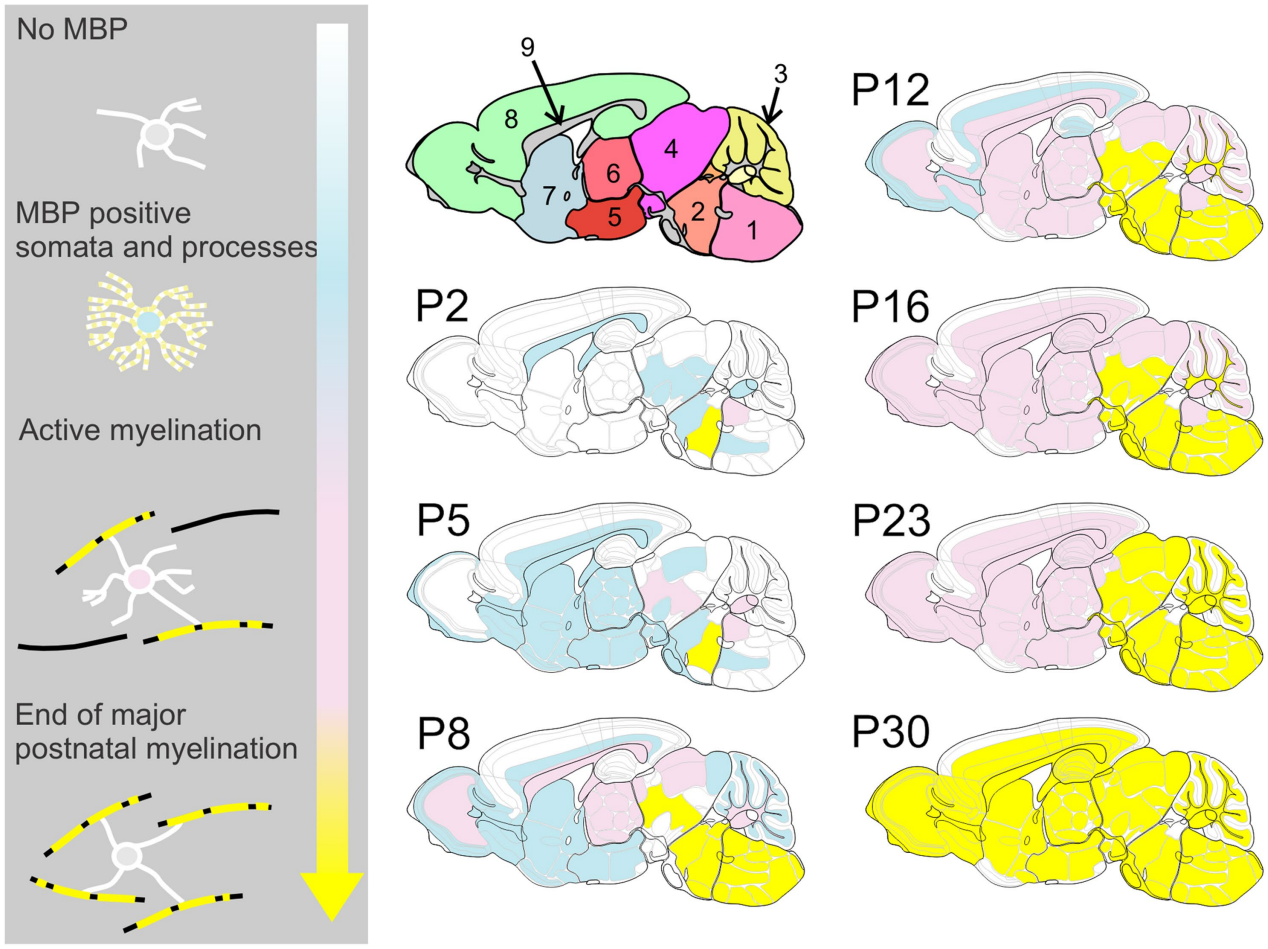
Spatiotemporal progression of MBP expression in the developing mouse brain. This schematic provides an overview of MBP labeling throughout brain development, highlighting the posterior-to-anterior and deep-to-superficial progression of myelination. Color Key – White: Regions with weak or absent MBP staining; Blue: MBP expression begins in oligodendrocyte cell bodies and processes, but no association with axons is yet observed; Pink: Active myelination is underway, with strong MBP staining associated with axons; Yellow: Regions where the most rapid phase of myelination has concluded. Brain regions in upper left cartoon: 1: medulla; 2: pons; 3: cerebellum; 4: midbrain; 5: hypothalamus; 6: thalamus; 7: cerebral nuclei; 8: cerebral cortex; 9: fiber tracts.

For space and clarity considerations, subsequent descriptions focus on MBP staining, though MAG staining data are available in the online dataset. Likewise, for each brain region, we present selected representative time points that span from initial MBP expression in cell somata and processes through completing major myelination events. Figure 5 provides a schematic overview of MBP labeling progression across development to help guide readers through the results.

### 3.3 Cerebellum

**Figure 6:**
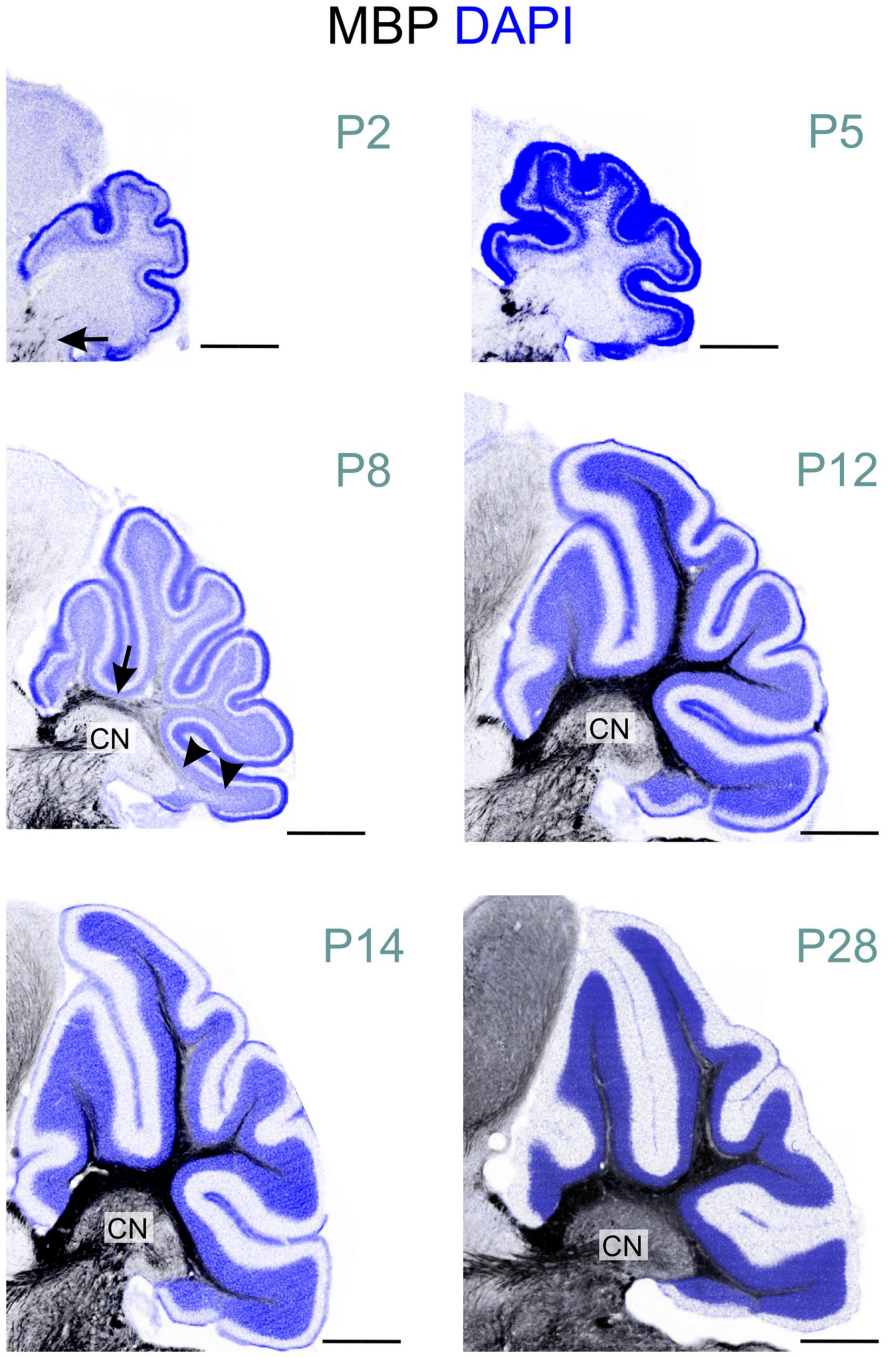
MBP staining in the developing cerebellum. Immunofluorescence staining for MBP (black) and cell nuclei (DAPI, blue) in the mouse cerebellum from postnatal P2 to P28. At P2, only a few short MBP-positive fibers are visible at the base of the cerebellar peduncle (arrow). By P8, intense MBP staining is visible at the base of the arbor vitae (arrow). In the posterior-ventral region, MBP- positive fibers extend to the lobule tips (arrowheads), while the anterior-dorsal area shows little to no staining. MBP-positive fibers are also visible in the cerebellar nuclei at this stage. At P12, intense MBP staining permeates the entire arbor vitae, reaching the tips of all lobules. Staining is also more pronounced staining in the cerebellar nuclei. From P12 to P28, MBP staining intensifies markedly throughout the cerebellum. Scale bars = 500 μm. CN, cerebellar nuclei.

**Figure 7:**
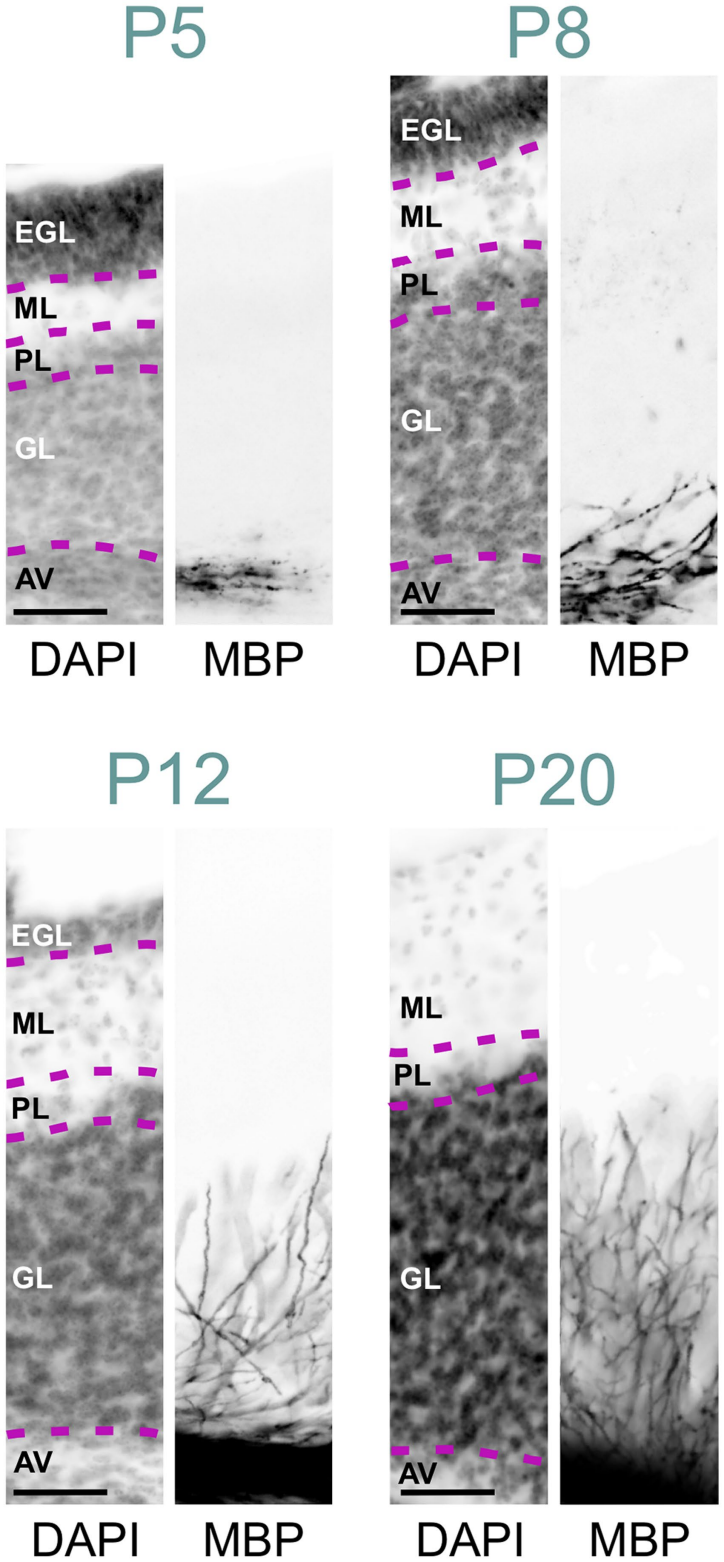
MBP staining in the developing cerebellar cortex. Immunofluorescence staining for cell nuclei (DAPI, left) and MBP (right) in the posterior- ventral region of the mouse cerebellar cortex from P5 to P28. At P5, a few MBP-positive fibers are visible in the arbor vitae, but the cerebellar cortex proper is unstained. By P8, more fibers in the arbor vitae show MBP staining, with a few penetrating the granule cell layer. At P12, intense MBP staining is present in the arbor vitae, with more positive fibers penetrating the granule cell layer and some reaching the Purkinje cell layer. At P20, the arbor vitae exhibit intense MBP staining, and numerous intensely positive fibers are visible throughout the granule cell layer. The molecular layer remains devoid of MBP staining. Scale bars = 50 μm. AV, arbor vitae; GL, granule cell layer; PL, Purkinje cell layer; ML, molecular layer; EGL, external granule cell layer.

At P2, a few oligodendrocytes and individual fibers at the base of the cerebellar peduncle were positive for MBP. Still, the cerebellum anlage showed no MBP immunoreactivity at that age (Figures 2, 6). By P5, a few MBP-positive fibers were visible in the arbor vitae (Figures 3, 6, 7). At P8, the arbor vitae displayed intensely stained fibers and fiber bundles. Staining density followed a distinct gradient: higher in the posterior-ventral region and lower in the anterior-dorsal area. In the most posterior-ventral region, MBP-positive fibers extended to the tips of the individual lobules, with some fibers extending just into the granule cell layer (Figure 7). Positive fibers were also visible in the cerebellar nuclei. By P12, the cerebellar nuclei showed intensified staining, while intense staining permeated the entire arbor vitae, reaching the tips of all lobules. The previously distinct small bundles of fibers merged into a uniform and homogeneously dense array of stained fibers. The granule cell layer showed increased penetration by positive fibers; in some lobules, these fibers extended to the Purkinje cell layer (Figure 7). Between P12 and P28, MBP staining intensified markedly throughout the cerebellum, particularly in the arbor vitae, cerebellar nuclei, and the granule layer of the cerebellar cortex. By P28, cerebellar MBP staining resembled that at P60.

### 3.4 Hindbrain

**Figure 8:**
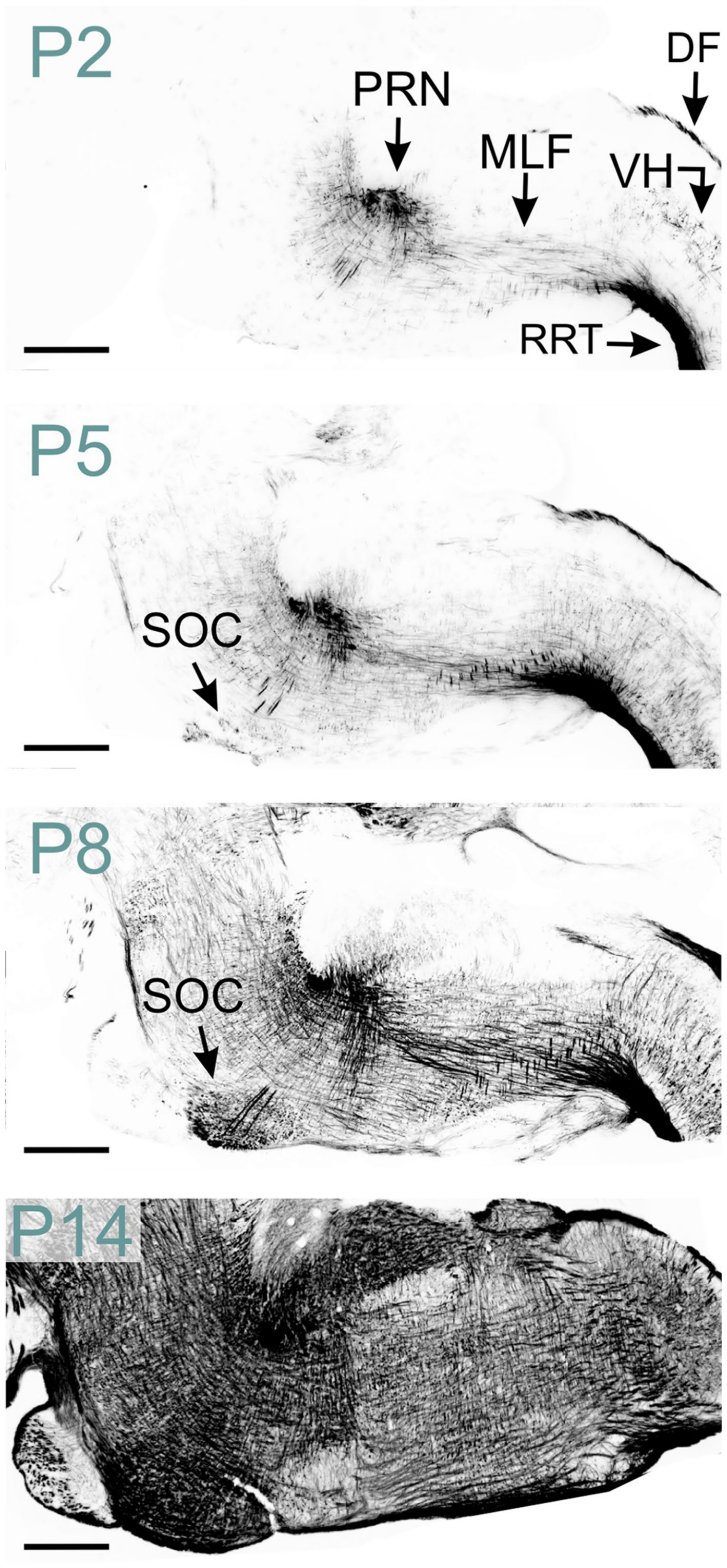
MBP staining in the developing hindbrain. Immunofluorescence staining for MBP in the mouse spinal cord and hindbrain from P2 to P14. At P2, strong MBP staining is observed in the spinal cord’s rostral reticulospinal tract and dorsal funiculus, as well as in the hindbrain’s pontine reticular nucleus and medial longitudinal fasciculus. MBP-positive fibers are also visible in the spinal cord’s ventral horn. By P5, MBP staining in the spinal cord’s ventral horn intensifies, and new MBP-positive areas emerge, including the superior olivary complex in the hindbrain. At P8, a strong and widespread MBP signal is observed throughout the hindbrain. Scale bars = 500 μm. DF, dorsal funiculus; MLF, medial longitudinal fasciculus; PRN, pontine reticular nucleus; RRT, rostral reticulospinal tract; SOC, superior olivary complex; VH, ventral horn.

MBP was detected in both white and gray matter of the spinal cord at P2, though gray matter exhibited significantly less staining (Figure 8). The rostral reticulospinal tract and dorsal funiculus showed intense MBP staining, while the ventral horn contained short, loosely arranged fibers. By P5, staining in the dorsal funiculus intensified, and the density of MBP-positive fibers in the gray matter increased. A marked expansion of MBP staining in the gray matter occurred by P14. By P60, gray and white matter displayed prominent, dense MBP staining (Figure 2).

In the hindbrain at P2, abundant MBP-positive fibers were evident in the pontine reticular nucleus and medial longitudinal fasciculus (Figures 2, 8). The reticular nuclei exhibited intensified MBP staining by P8, coinciding with the emergence of MBP staining in the superior olivary complex. A strong MBP signal was observed throughout all hindbrain nuclei by P14-P16. Subsequently, MBP levels continued to rise, albeit at a reduced rate.

### 3.5 Midbrain and interbrain

**Figure 9:**
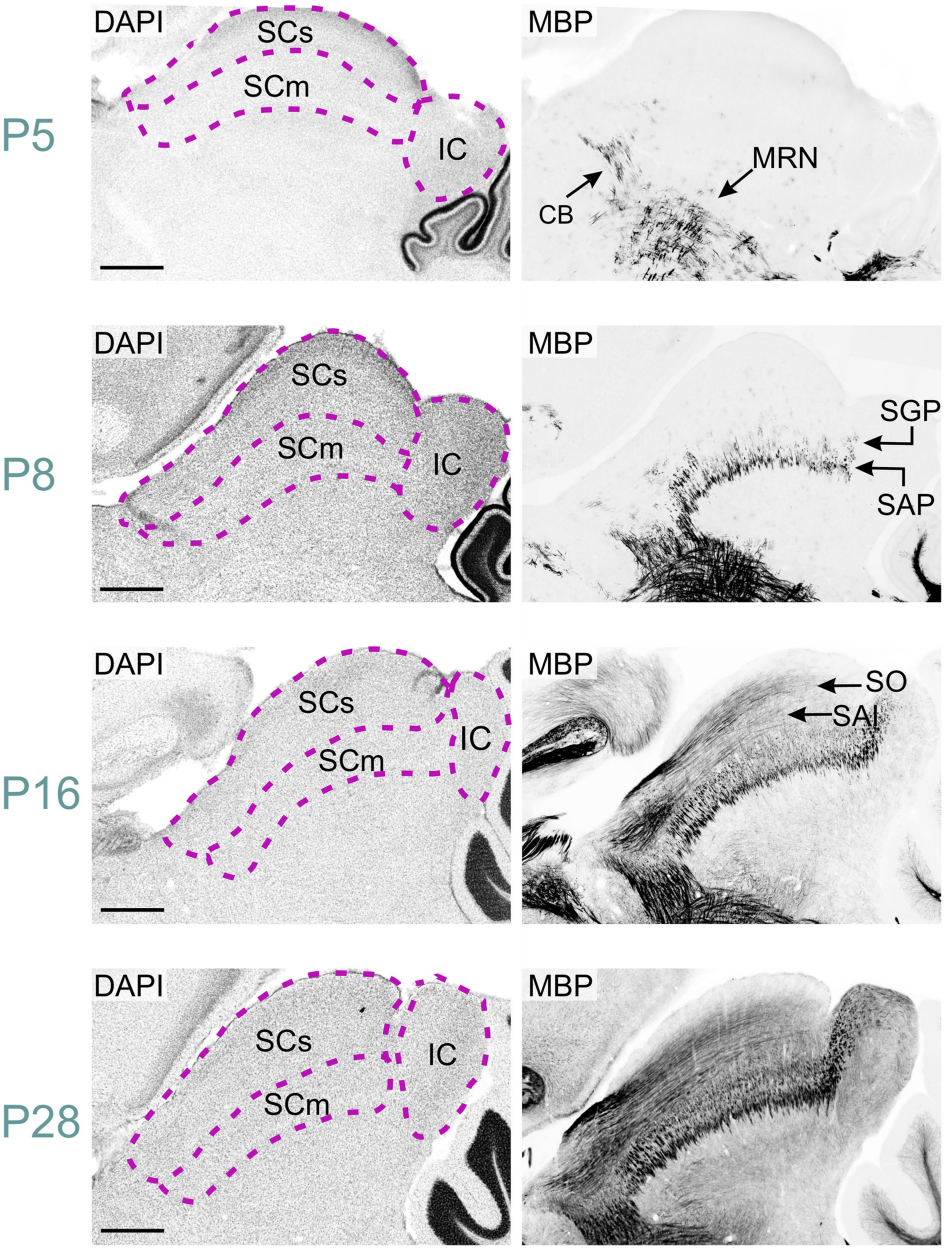
MBP staining in the developing midbrain. Immunofluorescence staining for DAPI (left panels) and MBP (right panels) in the mouse midbrain from P5 to P28. At P5, short MBP-positive fibers are visible in the midbrain reticular nucleus and cingulum bundle. By P8, these regions show longer fiber bundles, while short fibers appear in the stratum album profundum and stratum griseum profundum of the superior colliculus. At P16, long MBP-positive fibers are visible in the stratum opticum and stratum album intermediale of the superior colliculus, with also staining present in the inferior colliculus. By P28, MBP staining is widespread throughout the midbrain, with the strongest signal in the midbrain reticular nucleus, cingulum bundle, and colliculi. Scale bars = 500 μm. CB, cingulum bundle; IC, inferior colliculus; MRN, midbrain reticular nucleus; SAI, stratum album intermediale; SAP, stratum album profundum; SCm, superior colliculus motor-related; SCs, superior colliculus sensory-related; SGP, stratum griseum profundum; SO, stratum opticum.

We first detected MBP in the midbrain at P2. At this stage, scattered MBP-positive oligodendrocytes were seen across the entire midbrain reticular nucleus. By P5, numerous short MBP-positive fibers had appeared in the midbrain reticular nucleus and in the cingulum bundle (Figures 2, 9). MBP staining was also in a few scattered oligodendrocytes, especially in the motor region of the superior colliculus.

By P8, MBP staining had intensified in the midbrain reticular nucleus and cingulum bundle, with longer fiber bundles becoming apparent. The superior colliculus's motor region exhibited short fiber bundles, more prominent in the stratum album profundum compared to the stratum griseum profundum. MBP-positive oligodendrocytes emerged in the intermediate layer at this stage. Approximately three days post-eye-opening, at P16, staining further intensified in both the reticular nucleus and the superior colliculus's motor region. Long positive fibers appeared in the superior colliculus's sensory area, with greater density in the stratum opticum. The inferior colliculus also displayed numerous positive fibers at this point. MBP staining continued to intensify, albeit slower, from P16 to P28. By P28, the staining pattern closely mirrored that observed at P60.

**Figure 10:**
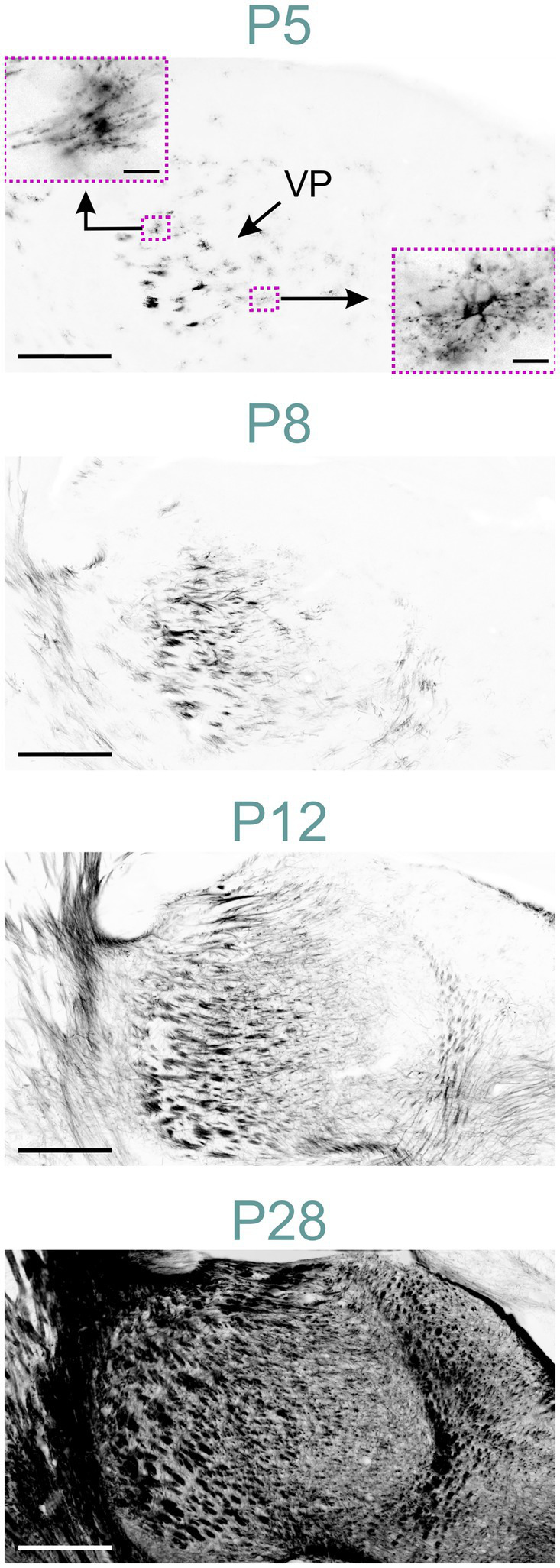
MBP staining in the developing thalamus. Immunofluorescence staining for MBP in the mouse thalamus P5 to P28. At P5, MBP is visible in oligodendrocytes throughout the thalamus, with a higher concentration in the ventral posterior nuclei. The lower right inset shows an oligodendrocyte with MBP located in radially extending processes and the soma. The upper left inset depicts an oligodendrocyte with polarized MBP staining concentrated in specific parts of the processes. By P8, individual fibers and short fiber bundles are visible in ventral posterior nuclei. At P12, MBP staining is present throughout the thalamus. By P28, intense MBP staining is observed in all thalamic nuclei. Scale bars = 400 μm; scale bars in inset = 20 μm. VP, ventral posteromedial nucleus.

At P5, MBP was detected in oligodendrocytes and their processes within the thalamus, primarily concentrated in the ventral posterior nuclei (Figure 10). Short fiber bundles emerged in these nuclei by P8. By P12, MBP-positive fibers were observed across all thalamic nuclei. The intensity and distribution of MBP staining progressively increased until P28, resulting in a robust MBP signal throughout the thalamus. After P28, staining continued to intensify, though at a reduced rate.

### 3.6 Cerebrum

**Figure 11:**
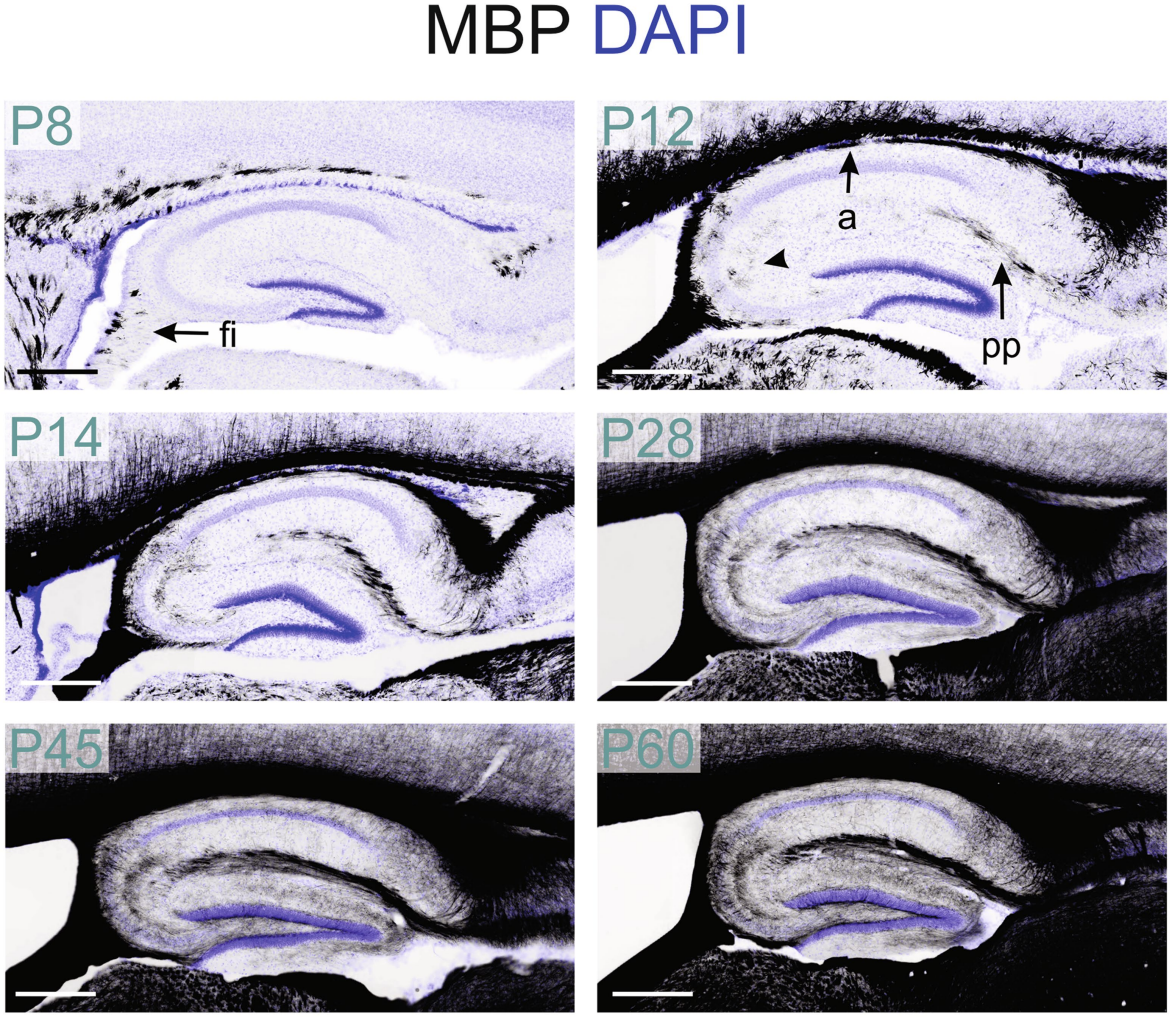
MBP staining in the developing hippocampal formation. Immunofluorescence staining for MBP (black) and DAPI (blue) in mouse hippocampal formation from P8 to P60. At P8, MBP-positive fibers are only in the fimbria. By P12, MBP staining is also present in the perforant pathway, and the alveus fibers and MBP-positive oligodendrocytes are visible in the CA2-3 region (arrowhead). From P14 to P28, positive fibers are visible across all hippocampal regions. From P28 to P60, MBP staining intensity and distribution continued to increase. Scale bars = 400 μm. a, alveus; fi, fimbria; pp., perforant pathway.

MBP-positive fibers first appeared in the hippocampal fimbria around postnatal P8 (Figures 2, 11). By P12, MBP staining intensified in the fimbria-fornix pathway, and fibers in the perforant pathway, located in the stratum lacunosum-moleculare, began to show positivity following a posterior-to-anterior gradient. MBP-positive oligodendrocytes and short fibers also emerged throughout the hippocampal formation, with a concentration in the CA2-3 fields.

**Figure 12:**
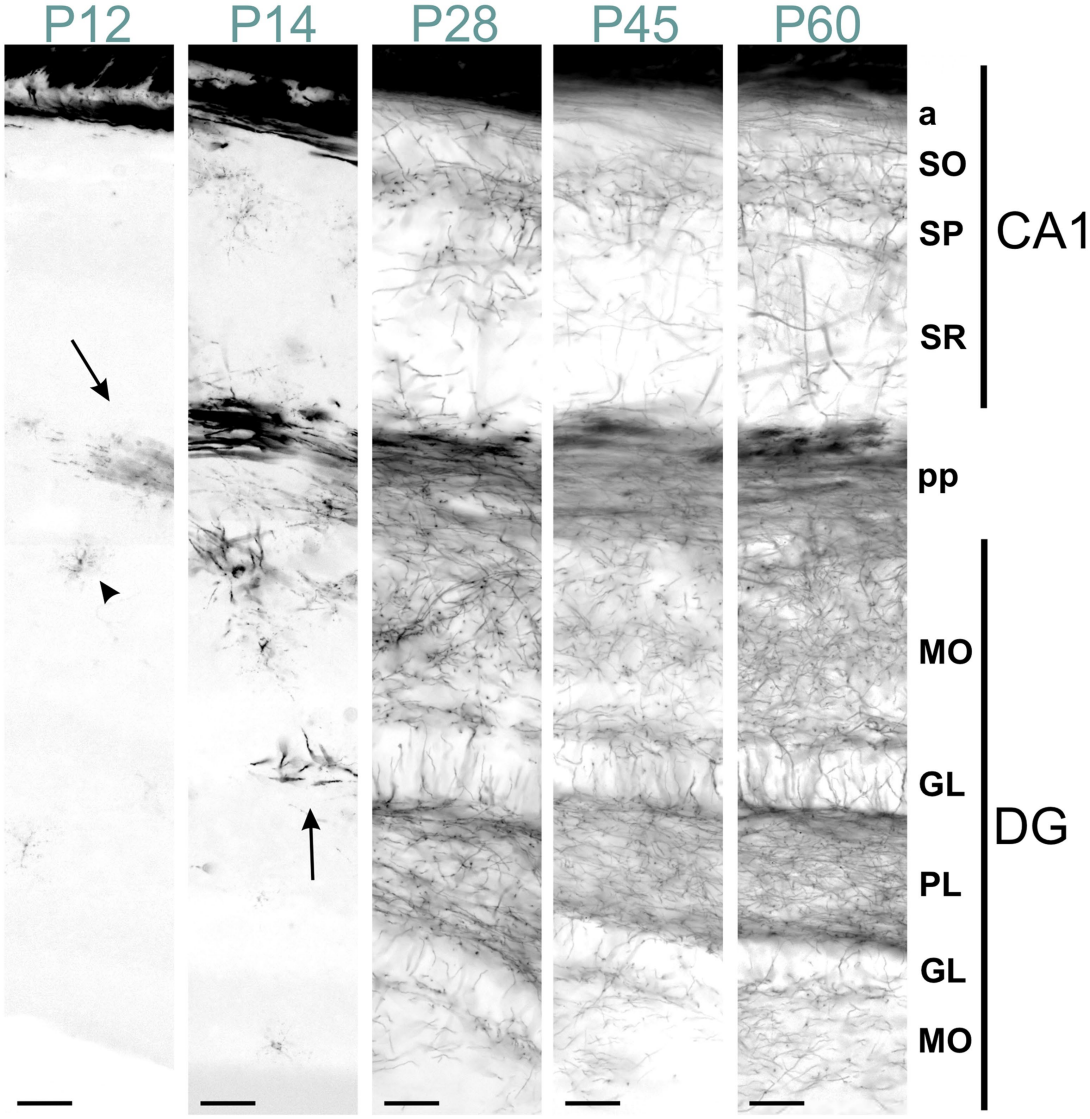
MBP staining in the developing CA1 field and dentate gyrus. Immunofluorescence staining for MBP across the CA1 field and dentate gyrus from P12 to P60. At P12, intense MBP staining is visible in perforant pathway fibers (arrow) and scattered oligodendrocytes (arrowhead). At P14, short MBP-positive fibers appear, especially in the dentate gyrus (arrow). From P28 to P60, MBP-positive fibers are visible in all CA1 and dentate gyrus layers, with progressively increasing density. In the stratum oriens, MBP-positive fibers on the neocortex side run parallel to the layer. In contrast, those closer to the pyramidal cell layer show a more network-like appearance. This network-like arrangement is also observed in the pyramidal cell layer and stratum radiatum. In the dentate gyrus, fiber density is highest in the polymorphic layer, followed by the molecular and granule cell layers. The polymorphic and molecular layers display a network-like organization, whereas in the granule cell layer, fibers are well-organized, crossing the layer perpendicularly. Scale bars = 50 μm. a, alveus; DG, dentate gyrus; GL, granule cell layer; MO, molecular layer; PL, polymorph layer; pp., perforant pathway; SO, stratum oriens; SP, stratum pyramidale; SR, stratum radiatum.

From P14 to P28, the MBP signal increased rapidly throughout the hippocampus, with the most intense staining observed in the fibers of the fimbria-fornix and perforant pathways. While positive fibers were present in all hippocampal regions, their distribution and orientation varied. The CA2-3 fields contained the highest density of positive fibers, with many extending across the stratum oriens to the stratum radiatum, perpendicular to these layers; these fibers likely belonged to the fimbria-fornix pathway. In contrast, the CA1 field showed only rare fibers extending across the stratum oriens to the stratum radiatum (Figure 12). Within the stratum oriens, MBP-positive fibers on the neocortex side tended to run parallel to the layer, while those closer to the pyramidal cell layer displayed a more “network” appearance with fibers at various angles. This network-like arrangement was also observed in the pyramidal cell layer and stratum radiatum. In the dentate gyrus, the densest concentration of fibers was seen in the polymorphic layer, followed by the molecular layer and the granule cell layer. The polymorphic and molecular layers exhibited a network-like organization of positive fibers, whereas in the granule cell layer, fibers were well-organized, crossing the layer perpendicularly. From there on, MBP staining intensity and distribution continued to increase until P60, but without organizational changes.

**Figure 13:**
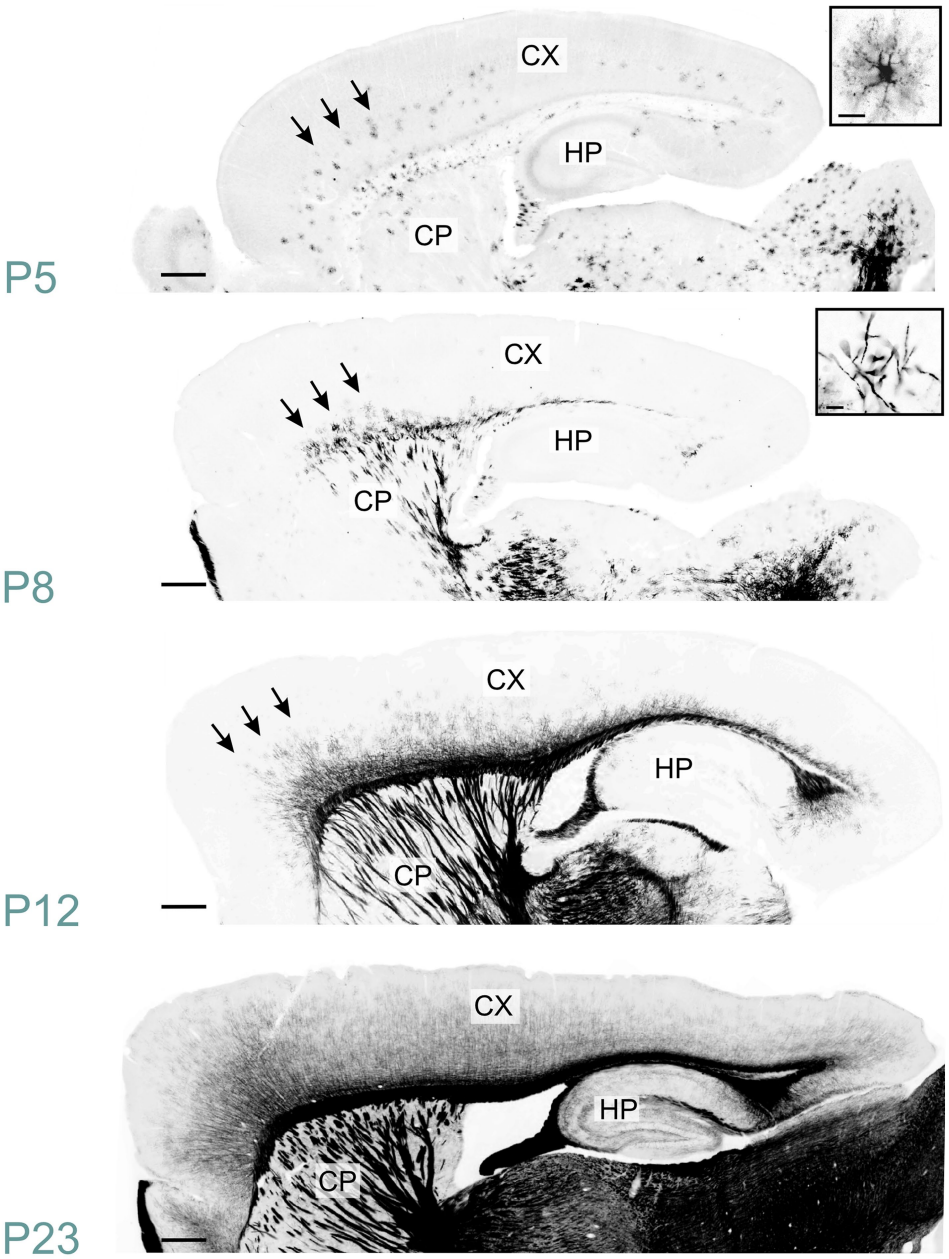
MBP staining in the developing cerebral cortex. Immunofluorescence staining for MBP in mouse neocortex from P5 to P23. At P5, MBP-positive oligodendrocytes (inset, top right) are scattered throughout the deep layers of the developing neocortex (arrows). By P8, short MBP-positive fibers (inset, top right) become visible in the deepest part of the neocortex, especially prominent in somatomotor areas (arrows). At P12, MBP-positive fibers have reached the middle layers in somatomotor areas (arrows). By P23, MBP-positive fibers extend across all cortical layers, though the density varies depending on the layer and cortical area. Scale bars = 400 μm. Inset scale bars = 20 μm. CP, caudoputamen; CX, Cerebral cortex; HP, hippocampal formation.

MBP immunostaining in the neocortex mirrored the cortex’s maturation pattern, advancing from deep to superficial layers (Figure 13). At P5, MBP staining was evident in oligodendrocyte somata and processes dispersed throughout layer 6. Scattered short positive fibers emerged in layer 6 by P8. P12 saw numerous longer MBP-positive fibers in layer 6, extending into layer 5. By P23, MBP-positive fibers extended across all cortical layers, appearing patchy in superficial layers. This patchiness was resolved by P45. The intensity and distribution of MBP staining continued to increase until P60.

MBP staining in the corpus callosum mirrored that of the corresponding neocortical regions (Figure 13). At P5, a few oligodendrocytes exhibited MBP positivity. MBP-stained fibers were visible throughout the corpus callosum by P12. However, the MBP signal continued to intensify until P60.

**Figure 14:**
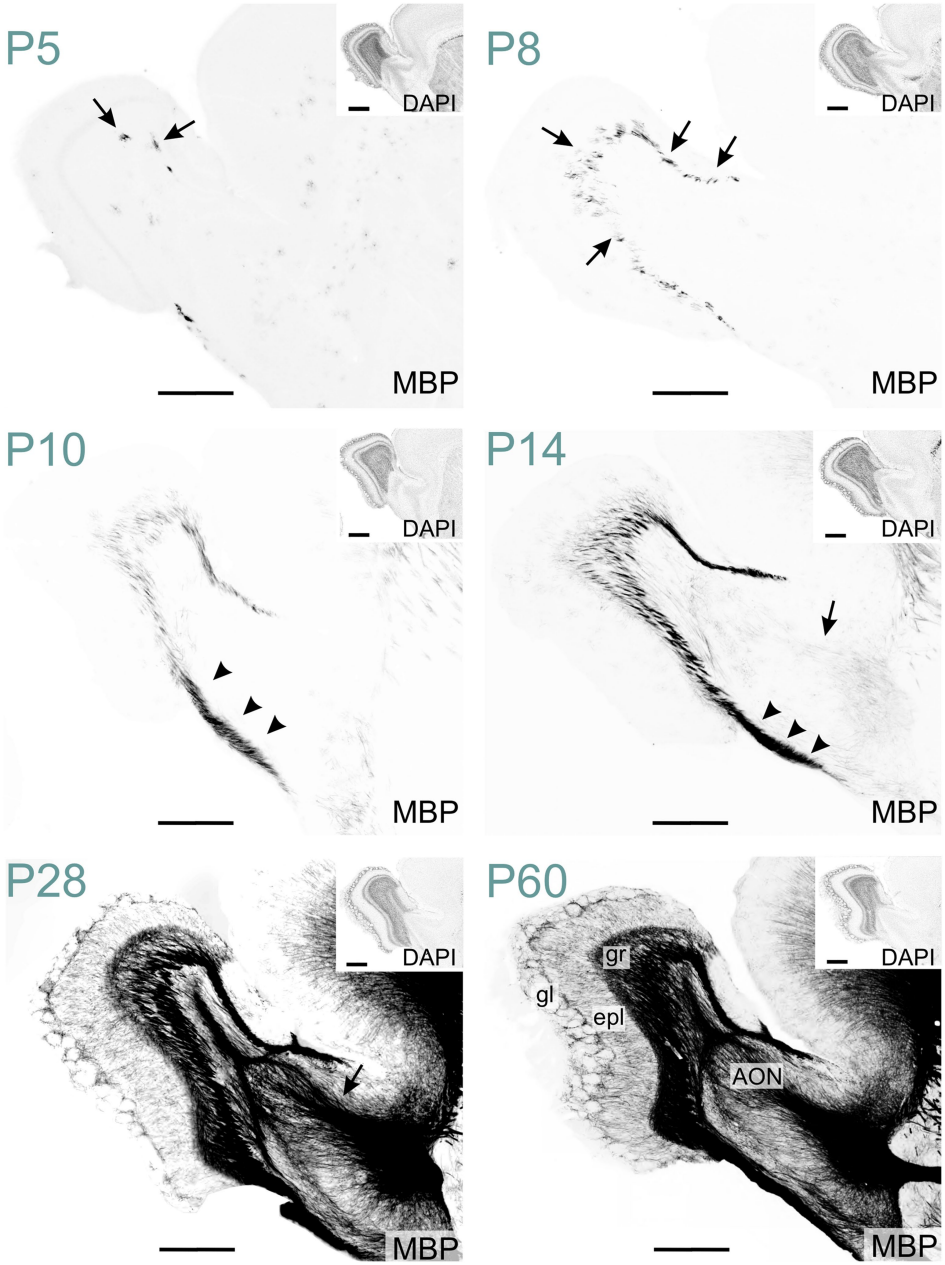
MBP staining in the developing olfactory regions. Immunofluorescence staining for MBP and nuclei (DAPI, inset on the top right) in the mouse olfactory regions from P5 to P60. At P5, MBP-positive oligodendrocytes are visible in the granule cell layer of the olfactory bulb (arrows). By P8, short, small MBP- positive fiber bundles appear throughout the granule cell layer (arrows). At P10, the lateral olfactory tract shows intense MBP staining (arrowheads). By P14, faintly MBP-positive fibers emerge in the anterior limb of the anterior commissure (arrow). At P28, MBP staining is present across all olfactory regions, with the strongest signal in the granule cell layer, lateral olfactory tract, and anterior commissure (arrow). By P60, MBP-positive fibers extend throughout the external plexiform layer and appear to encircle glomeruli. Scale bars = 500 μm. Inset scale bars = 500 μm. AON, anterior olfactory nucleus; epl, external plexiform layer; gl, glomerular layer; gr, granule layer.

In the olfactory regions, MBP was initially detected in the olfactory bulb. At P5, MBP-positive oligodendrocytes emerged in the olfactory bulb's granule cell layer, likely initiating the myelination of mitral cell axonal projections (Figure 14). By P10, the labeling in the granule cell layer had intensified. At this stage, the lateral olfactory tract exhibited clear staining, in contrast to the anterior limb of the anterior commissure, which remained mostly unstained. Faint fiber labeling in the anterior limb of the anterior commissure became visible around P14. By this age, the lateral olfactory tract was already strongly stained. At P28, MBP signal was seen across all olfactory bulb layers and piriform areas. The external plexiform layer and regions around glomeruli showed clear but sparse labeling. At that stage, the anterior olfactory nucleus displayed intense staining. MBP staining intensity and distribution continued to increase until P60. At this stage, dense labeling of fibers coursing through the granule cell layer was evident. Fibers were also seen throughout the external plexiform layer. MBP-positive fibers appeared to encircle the glomeruli.

### 3.7 Basal ganglia

**Figure 15:**
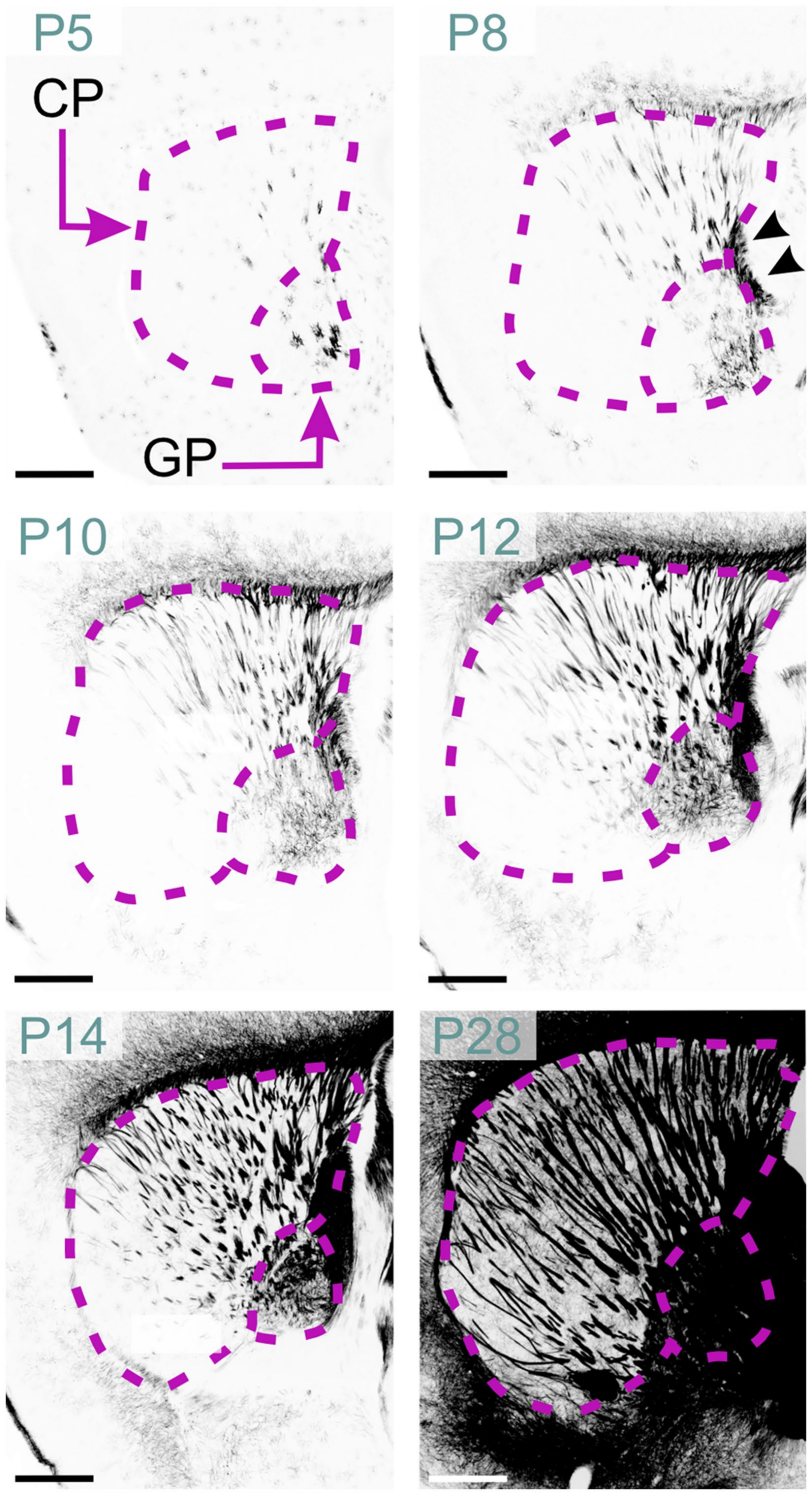
MBP staining in the developing basal ganglia. Immunofluorescence staining for MBP in the mouse basal ganglia from P5 to P28. At P5, oligodendrocytes are visible in the globus pallidus and caudoputamen. By P8, short and small fiber bundles appear in the caudal part of the caudoputamen, with individual fibers visible in the globus pallidus. Fibers in the internal capsule are also clearly stained (arrowheads). Between P10 and P28, MBP staining progresses rostrally to encompass the whole caudoputamen. By P28, intense MBP staining is present throughout the globus pallidus, caudoputamen, and internal capsule. Scale bars = 500 μm. CP, caudoputamen; GP, globus pallidus.

Scattered MBP-positive oligodendrocytes first appeared in the striatum at P2. By P5, they were also present in the globus pallidus (Figure 15). P8 marked the emergence of MBP-positive fiber bundles in the globus pallidus. Individually stained fibers and small bundles were also visible, penetrating the caudoputamen via the internal capsule. Short positive fibers also appeared in the external capsule at this stage. MBP staining intensity and distribution increased steadily up to P28 with densely stained fiber bundles in the globus pallidus, internal capsule, and external capsule. Sparse positive fibers were also evident in the striatal gray matter by P28. Post-P28, MBP signal continued to intensify, albeit at a reduced rate.

## 4 Discussion

To our knowledge, this study provides the most comprehensive immunohistochemical survey of MBP distribution in the developing mouse brain, with the resulting dataset made publicly accessible. While previous studies have examined MBP expression in specific brain regions or at limited developmental time points, our work offers a uniquely detailed spatiotemporal map of myelination across 13 developmental stages in the C57BL/6J mouse brain, including fiber-level resolution (0.3 μm/pixel), ~200 μm lateral sampling, more uniform staining across different brain structures, and a consistent protocol from tissue preparation through microscopy.

Furthermore, our optimized immunostaining protocol addresses the long-standing challenge of inconsistent MBP visualization, providing a more reliable and sensitive method for detecting subtle changes in myelination. We hope the comprehensive dataset, improved methodology, and open access availability position this study as a valuable resource for analyzing myelination progression throughout the mouse brain. The public dataset can serve as an important baseline for studies investigating myelination in various contexts, including genetic manipulations, disease models, and developmental disorders, ultimately facilitating a deeper understanding of both normal and pathological myelination processes.

### 4.1 Challenges in staining for MBP

Despite its abundance, MBP is challenging to stain consistently—an issue often overlooked in the literature. This difficulty likely arises from myelin’s unusual composition: approximately 80% lipid and 20% protein, which differs markedly from the typical 50:50 ratio in most biological membranes (Williams and Deber, 1993; Morell and Quarles, 1999). This lipid-rich structure can impede antibody penetration. Furthermore, myelin's lipid composition is distinctive, with higher proportions of cholesterol and glycolipids (40:40:20 cholesterol:phospholipid:glycolipid ratio) compared to typical biological membranes (25:65:10) (O’brien, 1965; Poitelon et al., 2020). The elevated cholesterol content may increase myelin's resistance to non-ionic detergents, potentially limiting the effectiveness of commonly used permeabilization agents like Triton X-100 in enhancing antibody penetration in myelin-rich tissue. This resistance to permeabilization, coupled with the wide range of myelinated fiber density across brain regions and developmental stages, complicates efforts to stain MBP consistently, making it challenging to establish a reliable correlation between actual MBP content and staining intensity across section thickness, brain areas, and time points. To address this problem, we adapted permeabilization techniques developed for light sheet microscopy (Renier et al., 2014), facilitating antibody diffusion using DMSO and Tween-20. We also found that 37°C incubation improved staining quality compared to 4°C or room temperature. This improvement likely stems from increased antibody specificity rather than enhanced penetration, aligning with the Arrhenius equation and the exothermic nature of antibody-antigen binding. At higher temperatures, the dissociation constant increases disproportionately compared to the association constant, effectively lowering the equilibrium constant. While initial binding occurs more readily at 37°C, only the highest-affinity interactions persist, resulting in reduced background and improved signal-to-noise ratio. Combining enhanced permeabilization with temperature-dependent selectivity, this optimized protocol enabled reliable staining across diverse brain structures and developmental ages.

Despite our improved protocol, MBP staining remains limited to tissue sections, providing only 2D representations of complex 3D myelin architecture. This limitation particularly affects our understanding of regions with intricate fiber organization. While our high-resolution images capture detailed myelin structures within individual sections, they cannot fully reveal the three-dimensional complexity of myelin networks. Future advances in whole brain 3D imaging techniques, such as light sheet microscopy combined with magnetic resonance microscopy, may help overcome this limitation.

### 4.2 Translational considerations

Translating developmental data from rodent models to humans presents significant challenges, reflecting their evolutionary divergence approximately 85 million years ago. Despite fundamental similarities in myelination processes, important species differences must be considered when applying mouse research findings to humans.

Temporal differences in myelination are particularly striking. For example, humans show substantial myelination in peripheral nerves, pons, and cerebellar peduncles at birth, while mice show limited myelination at this stage. While mice complete major myelination events within weeks, human myelination extends over years and continues into early adulthood, particularly in higher-order brain regions like the prefrontal cortex (Semple et al., 2013; Fletcher et al., 2021; Zeiss, 2021; Khelfaoui et al., 2024).

Adult myelination patterns also show species-specific features. Humans demonstrate remarkable myelin plasticity, with significant remodeling possible in adulthood (Yeung et al., 2014), particularly during skill acquisition (Scholz et al., 2009). At the molecular level, recent oligodendroglial mRNA and CNS myelin proteome analyses reveal that while myelin composition is largely conserved between species, certain proteins are unique to humans or mice (Gargareta et al., 2022). Therefore, the myelination patterns described in this study must be interpreted carefully when extrapolating them to humans.

### 4.3 Research and medical importance of MBP visualization

MBP immunohistochemistry serves as a key method for studying disorders affecting myelination. These disorders encompass a broad spectrum of conditions, including acquired and genetic disorders. One major category of acquired disorders involves prenatal exposure to substances that disrupt myelination. Prenatal alcohol exposure, a leading preventable cause of neurodevelopmental abnormalities in the United States (Popova et al., 2023), is associated with delayed development and myelination of white matter tracts, potentially resulting in persistent neurocognitive and behavioral disabilities (Fortier et al., 2014; Zahr and Pfefferbaum, 2017; Noor and Milligan, 2018; Crespi et al., 2020). Other substances, notably opioids, can also disrupt myelination (Merhar et al., 2019). Developmental hypoxia and inflammation can also disrupt myelination, causing newly formed oligodendrocyte precursor cells to struggle in maturing into myelinating oligodendrocytes (Back, 2017; He et al., 2022). This results in diffuse white matter injuries, the primary form of damage in premature birth survivors. These injuries can cause various neurological complications, including audiovisual dysfunction and cognitive impairment. The severe outcome is cerebral palsy, the most common motor disability in childhood (Jiang et al., 2020).

Genetic disorders also affect myelination. They fall into two main groups. The first includes mutations in genes involved in myelin production or maintenance, resulting in hypomyelinating leukodystrophies. These are characterized by reduced myelin formation, developmental delay, hypotonia, spasticity, and variable intellectual disability (Wolf et al., 2021). The second group comprises various genetic disorders where delayed or abnormal myelination appears as a secondary feature. This includes specific intellectual disabilities like Angelman syndrome and Pitt-Hopkins syndrome, as well as autism spectrum disorder (Pacey et al., 2013; Galvez-Contreras et al., 2020; Phan et al., 2020; Petazzi et al., 2023). Mouse models are extensively used to study this plethora of myelination disorders. MBP immunohistochemistry is one of the essential methods for examining differences in myelination patterns and evaluating therapeutic interventions. Many of these studies require detailed reference data on typical myelination trajectories to optimize research efficiency and resource allocation. Such baselines are important for identifying the most relevant brain regions and developmental time points to investigate. With this report and the accompanying high-resolution image dataset, we aim to address these needs and facilitate research into myelination processes and related disorders. Beyond serving as a reference, this dataset enables quantitative analyses and reuse of the imaging data, following established practices in genomics research. Its public accessibility through standardized platforms supports the growing adoption of shared resources in neuroscience, facilitating collaborative research and accelerating discoveries.

## Data Availability

The datasets presented in this study can be found in online repositories. The names of the repository/repositories and accession number(s) can be found below: BioImage Archive and Image Data Resource (accession S-BIAD1483, DOI: 10.6019/S-BIAD1483).

## Footnotes

^1^ https://idr.openmicroscopy.org/

## Glossary

a: Alveus
AON: Anterior olfactory nucleus
CA1: CA1 field
CB: Cingulum bundle
CC: Corpus callosum
CN: Cerebellar nuclei
CP: Caudoputamen
CX: Cerebral cortex
DF: Dorsal funiculus
DG: Dentate gyrus
EGL: External granule cell layer
epl: External plexiform layer
FBST: PBS with 10% fetal bovine serum and 0.2% Triton-X-100
fi: Fimbria
gl: Glomerular layer
GL: Granule cell layer
GP: Globus pallidus
gr: Granule layer
HP: Hippocampal formation
IC: Inferior colliculus
MAG: Myelin-associated glycoprotein
MBP: Myelin basic protein
ML: Molecular layer of cerebellum
MLF: Medial longitudinal fasciculus
MO: Molecular layer of dentate gyrus
MRN: Midbrain reticular nucleus
PBS: Phosphate-buffered saline
PBST: PBS with 0.2% Triton-X-100
PL: Polymorph layer
PL: Purkinje cell layer
pp.: Perforant pathway
PRN: Pontine reticular nucleus
RRT: Rostral reticulospinal tract
SAI: Stratum album intermediale
SAP: Stratum album profundum
SCm: Superior colliculus motor-related
SCs: Superior colliculus sensory-related
SO: Stratum opticum
SO: Stratum oriens
SOC: Superior olivary complex
SP: Stratum pyramidale
SR: Stratum radiatum
ST: Striatum
VH: Ventral horn
VP: Ventral posteromedial nucleus
